# Role of EphB2/ephrin-B1 signalling in the development and progression of obesity-associated colorectal cancer

**DOI:** 10.3892/ol.2022.13436

**Published:** 2022-07-19

**Authors:** Yoshiyuki Suzuki, Koji Okabayashi, Hirotoshi Hasegawa, Masashi Tsuruta, Ryo Seishima, Toshiki Tokuda, Yuko Kitagawa

**Affiliations:** Department of Surgery, Keio University School of Medicine, Tokyo 1608582, Japan

**Keywords:** inflammation, colon cancer, carcinogenesis, obesity-associated cancer, ephrin

## Abstract

Obesity is a major problem worldwide and has been associated with colorectal cancer development, among other diseases. Ephrin receptors and ligands play an important role in the turnover of the intestinal mucosa and intestinal crypt compartmentalization. It has been hypothesised that obesity-induced inflammation affects ephrin signals, leading to carcinogenesis. Therefore, the aim of the present study was to assess the relationship between Eph-ephrin B signalling, obesity and obesity-associated colorectal cancer. An azoxymethane-induced obesity-associated cancer KKAy mouse model developed in our prior study was used. A total of 46 patients with consecutive colorectal cancer and 48 tumours were analysed. Immunohistological analyses were performed in mouse and human samples, and immunoreactive scores (IRS) were determined. KKAy mice were significantly more prone to cancer development compared with control C57/BL mice (2/15 in C57/BL vs. 10/10 in KKAy; P<0.001). TUNEL assay revealed a lower number of apoptotic cells in normal mucosa of KKAy mice (8.8% in C57/BL vs. 3.2% in KKAy; P<0.001) and obese patients (9.2% with BMI <25 vs. 3.6% with BMI ≥25; P=0.021). Immunohistological analysis revealed that ephrin-B1 was downregulated in normal mucosa from KKAy mice and obese patients (IRS, 2.86 with BMI <25 vs. 6.00 with BMI ≥25; P=0.002). Moreover, EphB2 was downregulated in tumours from KKAy mice and obese patients (IRS, 6.58 with BMI <25 vs. 3.83 with BMI ≥25; P<0.001). The distribution of infiltrated macrophages corresponded to the MCP-1 expression pattern in KKAy mice, and the number of macrophages was also significantly higher in those mice (36.3 in C57/BL vs. 120.0 in KKAy; P=0.029). The findings suggested that obesity results in disruption of EphB2/ephrin-B1 signalling, promoting colorectal cancer development and progression.

## Introduction

The prevalence of excess body weight and obesity is rapidly increasing. Recent studies have shown that the number of obese adults has reached more than 600 million worldwide in 2014 (1). Excess body weight and obesity are well-known risk factors for cardiovascular diseases and diabetes (2,3). Additionally, several cohort studies have shown that excess body weight and obesity are associated with cancer development. Colorectal cancer is the third most common cancer worldwide, and approximately 8% of colorectal cancer cases can be attributed to obesity (4,5). Thus, obesity-associated colorectal cancer imposes serious challenges to healthcare systems globally. A previous meta-analysis revealed that higher body mass index (BMI) was a significant risk factor for colorectal adenomas (6); another large cohort study suggested that higher BMI was an independent risk factor for colorectal adenoma incidence and adenoma recurrence (7).

The intestinal mucosa is composed of crypts and finger-like protrusions (villi). The crypt bottoms contain rapidly proliferating intestinal stem cells and transit-amplifying progenitor cells. These cells differentiate into enterocytes, goblet cells, or enteroendocrine cells, and migrate as clonal lineages to the villi within 4–5 days, where they are shed into the lumen (8). The intestinal stem cell differentiation, also known as crypt-villus axis, is strictly regulated, and dysregulation of this process is considered to play a crucial role in colon carcinogenesis (8,9). Ephrin receptors and ligands are essential in the crypt-villus axis regulation and have been, therefore, implicated in colorectal cancer.

Ephrin receptors are the largest family of receptor tyrosine kinases (RTKs). Their ligands, ephrins, are membrane-anchored proteins and are divided into two subclasses: type-A ephrins, and type-B ephrins. EphA receptors (EphA1-EphA10) bind type-A ephrins, while EphB receptors (EphB1-EphB6) bind type-B ephrins. Eph/ephrin signalling regulates tissue and organ patterning, in addition to controlling cell survival during normal and neoplastic development (10). Moreover, EphB/ephrin-B signalling plays a critical role in the turnover of the intestinal mucosa by controlling the cell positioning along the intestinal crypt (8,9). Abnormal ephrin expression has been implicated in a variety of human cancers, including colorectal cancer (11–13). Recent studies have suggested that ephrins may also play a tumour-suppressive role in some cancers, highlighting the complex role of Ephs in oncogenesis (9,14–16). Previous studies have demonstrated that colorectal cancer development and progression were suppressed by the repulsive interactions between ephrin-B1-positive normal epithelial cells and EphB-positive tumour cells (9,14,15,17).

Interestingly, a few studies have suggested an association between ephrins and obesity. It has been shown that obesity-related inflammatory cytokines induce the downregulation of ephrin-B1 in adipose cells (18), suggesting that obesity not only promotes the secretion of cytokines but also influences the structure of the gastrointestinal mucosa through cytokine secretion. As the majority of studies have focused on the relationship between cytokine secretion and obesity-related colorectal carcinogenesis, little is known about colonic mucosal changes that happen during obesity-related colorectal cancer development and progression. Considering the key role of crypt-villus axis dysregulation in colorectal carcinogenesis, investigating the association between obesity-related colorectal cancer development and alterations in the crypt-villus axis is of great importance.

The aim of this study was to elucidate the association between obesity-associated colorectal cancer and alterations in the crypt-villus axis induced by Eph/ephrin signalling, which will allow for the establishment of preventive measures for obesity-associated cancer.

## Materials and methods

### Mouse experiments

**Figure 1.**
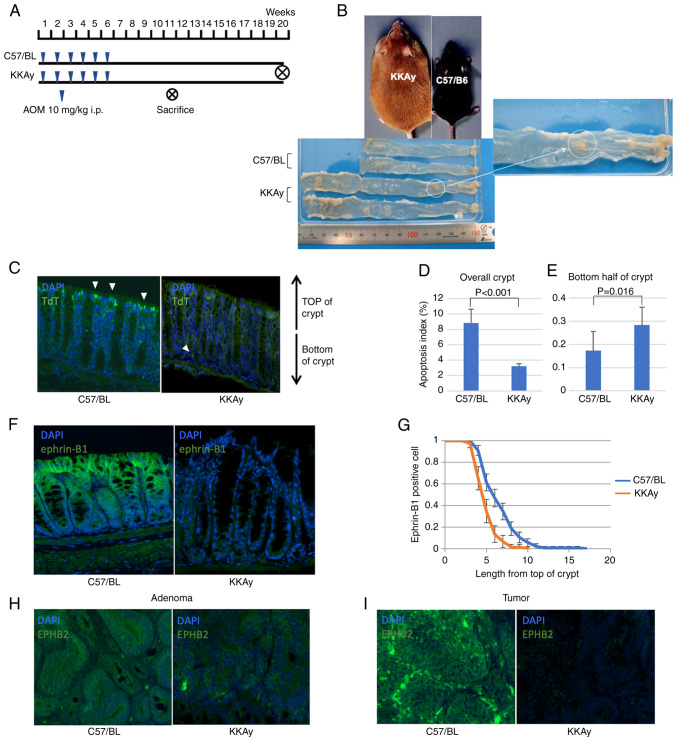
TdT-mediated dUTP nick-end labelling, and expression analysis of ephrin-B1 and EphB2 in murine colon. (A) Procedure of the azoxymethane-induced obesity-associated cancer model. (B) Macroscopic images of colons of C57/BL and KKAy mice. (C) Representative fluorescent TUNEL images of normal mucosa from C57/BL and KKAy mice. White arrows indicate apoptotic cells. Magnification, ×200. (D) Apoptosis index in the whole crypt of C57/BL and KKAy mice. (E) Apoptosis index in the bottom half of the crypt of C57/BL and KKAy mice. (F) Representative immunohistochemistry images of the normal, ephrin-B1 stained mucosa of C57/BL and KKAy mice. Magnification, ×200. (G) Analysis of ephrin-B1-positive cell position in the crypt of C57/BL and KKAy mice. (H) Representative immunohistochemistry images of C57/BL and KKAy mouse adenoma after EphB2 staining. Magnification, ×200. (I) Representative immunohistochemistry images of C57/BL and KKAy mouse tumours after staining for EphB2. Magnification, ×200. Error bars indicate 95% confidence intervals. TdT, terminal deoxynucleotidyl transferase; dUTP, deoxyuridine triphosphate; C57BL, C57BL/6JJcl mice; KKAy, C57BL/6JJcl-derived KK-Ay/TAJcl mice.

Fifteen male C57BL/6JJcl mice (C57BL mice) and fifteen male KK-A^y^/TAJcl mice (KKAy mice) were purchased from Nippon CLEA (Tokyo, Japan). KK mice are polygenic mouse models of type 2 diabetes mellitus; leptin and its receptor are intact in these animals (19). The genes responsible for the inherited characters of KK are unknown. KKAy mice were obtained by cross-mating KK mice with C57BL mice with type 2 diabetes mellitus but normal leptin and leptin receptor levels and 6J-Ay carrying the Agouti yellow (Ay) gene. The latter mice exhibit severe hyperphagia, impaired glucose tolerance, and hyperlipidaemia; such mice are often used in studies on metabolic disease (20). Five-week-old male C57BL and KKAy mice were intraperitoneally injected with a single dose of 10 mg/kg (approximately 200 µg/mouse) azoxymethane (AOM) (Sigma-Aldrich, St. Louis, MO) weekly for 6 weeks. Two groups of mice were culled at the 20th week (Fig. 1A). Euthanasia involved cervical dislocation; death was confirmed by cardiac and respiratory arrest. All animal procedures were performed in accordance with our institutional guidelines and were approved by the ethics committee of Keio University School of Medicine Ethics Committee (approval no. 20140001).

### Patients and clinical samples

The expression levels of Ephrin receptors and ligands were analysed in a panel of human benign and colorectal cancer (CRC) specimens resected from colorectal cancer patients. Consecutive patients who underwent surgical resection of stages I–IV CRC at Keio University Hospital between September 2015 and March 2016 were enrolled. The study included patients with pathologically confirmed American Joint Committee on Cancer (AJCC) stage I–IV CRC who underwent resection of the primary lesions. The study excluded patients with ulcerative colitis-associated cancer, familial adenomatous polyposis, limited resection (such as trans-anal resection), or unavailable data. The clinical characteristics and pathological findings of the patients, including sex, BMI, degree of tumour differentiation, and AJCC stage (TNM stage), were collected from medical records. Certificated pathologists assessed the degree of tumour differentiation in colorectal cancer according to AJCC guidelines (21). Patients with a BMI higher than 25 kg/m^2^ were defined as obese. Fifty primary CRCs from patients who underwent colectomy as primary treatment, as well as paired normal colon mucosa samples were collected during the operation at Keio University Hospital (Tokyo, Japan) between September 2015 and March 2016. Patient consent, including for the use of tumour tissue, was obtained using the opt-out method. The study was approved by the Keio University School of Medicine Ethics Committee (approval number: 20140001).

### TdT-mediated dUTP nick-end labelling (TUNEL) staining

For the TUNEL staining, tissue slides were deparaffinised, rehydrated, and digested with 1 µg/ml proteinase K for 10 min at 37°C. Next, TUNEL staining was performed using the ApopTag^®^ Fluorescein *In Situ* Apoptosis Detection Kit (Merck Millipore, Darmstadt, Germany) according to the manufacturer's instructions. Then, the samples were mounted with DAPI Vectashield (Vector Laboratories, Burlingame, CA, USA). The frequency of apoptosis was expressed as the apoptotic index, which was calculated as the apoptotic cell count in a crypt divided with the total cell count of the crypt.

### Immunohistochemistry (IHC)

Paraffin-embedded sections (5m) were deparaffinised with xylene and rehydrated with a graded series of ethanol. Sections were incubated with 2.0% hydrogen peroxide for 30 min to block endogenous peroxidase. Antigen retrieval was carried out by boiling in 10 mM sodium citrate buffer. The following antibodies were used: goat anti-EphB2 (1:200; R&D systems, Minneapolis, MN, USA), goat anti-ephrinB1 (1:400; R&D systems), rat anti-Macrophage (1:30; BMA Biomedicals, Rheinstrasse, Switzerland), goat anti-monocyte chemoattractant protein-1 (MCP-1) (1:100; R&D Systems), and rat anti-diphosphorylated extracellular signal-regulated kinase 1/2 (ERK1/2) (1:100; Sigma-Aldrich, St. Louis, MO, USA). The sections were stained with haematoxylin-eosin (HE) and observed under a light microscope. For fluorescent IHC, samples were stained for ephrin-B1- and EphB2-GFP and mounted using DAPI Vectashield.

To assess the immunoreactivity, the sections were scored in terms of their proportion (score 0,-10%; 1, 10–50%; 2, 50–80%; 3, >80%) and intensity (score 0: none; 1, weak; 2, moderate; 3, strong) as previously reported (22); the scoring was performed by two investigators (Y.S. and T.T.) who were blinded to the clinicopathological factors. The total score was calculated by multiplying the two scores.

### Statistical analyses

Continuous variables were expressed as averages and standard error. Associations between Eph-ephrin expression and other variables were analysed using Pearson's chi-squared test, Fisher's exact probability test, the Mann-Whitney U test or Kruskal-Wallis test followed by Dunn's post hoc test. All analyses were two-sided, and P-values <0.05 were considered statistically significant. Statistical analyses were performed using SPSS ver. 23 (IBM Corp, Armonk, NY, USA).

## Results

### Obesity and colorectal carcinogenesis

The incidence of colorectal cancer in KKAy mice and C57BL mice was assessed using an AOM-induced colon carcinogenesis mouse model. Fig. 1B shows colonic macroscopic images of both mouse strains. KKAy mice were heavier and had longer intestines than C57BL mice. Even though only two of 15 C57BL mice developed colorectal tumours, tumourigenesis occurred in all KKAy mice (P<0.001, Mann-Whitney U test). The average tumour diameter was larger in KKAy mice compared with C57BL mice (largest tumour diameter: 0.47±1.36 mm in C57BL vs. 7.40±5.70 mm in KKAy mice; P<0.001, Mann-Whitney U test; Table I). These results suggested that obesity enhanced CRC development.

### Dysregulation of apoptosis in obese mice

To assess the influence of cell turnover dysregulation in the gastrointestinal mucosa, the frequency and localisation of apoptotic cells in colon mucosa were compared between C57BL mice and KKAy mice (Fig. 1C). Generally, intestinal cell apoptosis occurs on the luminal side of the crypt, resulting in mucosal shedding. Apoptosis in normal colon mucosa is significantly suppressed in KKAy mice; the apoptotic index in C57BL mice was 8.8%, while in KKAy it was only 3.2% (P<0.001, Mann-Whitney U test; Fig. 1D). Interestingly, apoptotic cell death was more frequently observed at the base of the crypt in KKAy mice than in C57BL mice (apoptotic index in the bottom half of the crypt: 0.17% in C57BL vs. 0.28% in KKAy, P=0.016, Mann-Whitney U test; Fig. 1E), which suggested the dysregulation of cell turnover in KKAy mice.

### Expression of EphB2 and ephrin-B1 in murine colon

Typically, ephrin-B1 is strongly expressed in cells at the top of the crypt, while its expression is very low at the bottom of the crypt. The expression patterns of EphB2 and ephrin-B1 in the intestinal mucosa of C57BL and KKAy mice were compared, and it was found that ephrin-B1 was expressed in lower levels in KKAy mice than in C57BL mice (Fig. 1F). Additionally, ephrin-B1 positive cells were frequently observed in the crypts of C57BL mice (Fig. 1G). Although EphB2 expression was detected at the bottom of the crypts in C57BL and KKAy mice, it was expressed at lower levels in KKAy mice compared with C57/BL mice. Interestingly, in KKAy mice, EphB2 was expressed at lower levels in adenomas, and its expression was not detectable in cancer (Fig. 1H and I). In contrast, in C57BL mice, EphB2 was expressed in normal mucosa, adenoma and cancer.

### Expression of MCP-1 and macrophage migration in murine colon

**Figure 2.**
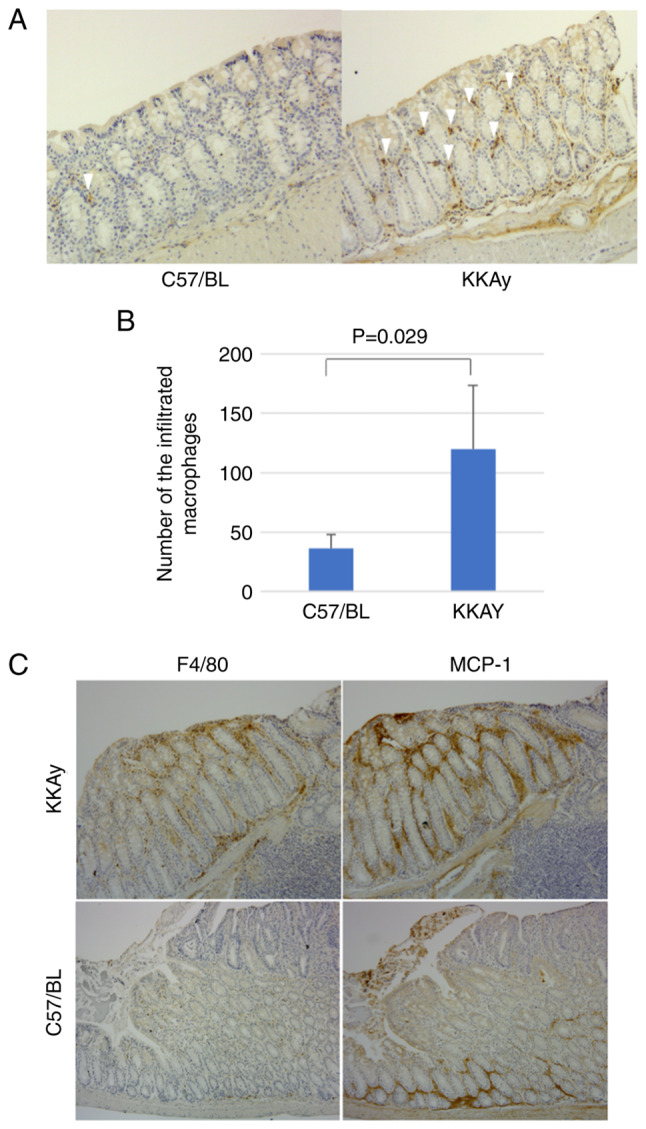
Macrophage infiltration and MCP-1 expression in murine colon lamina propria. (A) Representative immunohistochemistry images of normal, F4/80-stained mucosa from C57/BL and KKAy mice. Magnification, ×100. (B) Comparison of the number of infiltrating macrophages in the mucosa of C57/BL and KKAy mice. (C) Representative immunohistochemistry images showing MCP-1 and F4/80 expression in the same region of consecutive slices from C57/BL and KKAy mice. Magnification, ×100. Error bars indicate 95% confidence intervals. MCP-1, monocyte chemoattractant protein-1; C57BL, C57BL/6JJcl mice; KKAy, C57BL/6JJcl-derived KK-Ay/TAJcl mice.

In normal colon mucosa, the number of macrophages in C57/BL mice is significantly lower than in KKAy mice (36.3±11.9 in C57/BL vs. 120.0±54.8 in KKAy, P=0.029, Mann-Whitney U test; Fig. 2A and B). To investigate the relationship between inflammation and alterations in the crypt-villus axis, the expression of MCP-1 was evaluated. A previous study reported that MCP-1 downregulated ephrin-B1 expression in adipose cells (18). Initially, our hypothesis was that MCP-1 expression is regulated by ephrin-B1 in the intestinal epithelium. Ephrin-B1 promotes macrophage migration in the lamina propria of the murine colon. Fig. 2C shows MCP-1 and F4/80 expression in the same region in consecutive slices. MCP-1 expression was elevated around the tumour and adenoma in both C57/BL and KKAy mice. The distribution of infiltrated macrophages corresponded to the MCP-1 expression pattern in KKAy mice; however, C57/BL mice had a lower number of infiltrated macrophages, and their distribution did not correspond to the MCP-1 expression pattern. These results suggest that obesity accelerated the inflammation of colon mucosa through MCP-1 upregulation and subsequent macrophage infiltration.

### ERK1/2 expression in the murine colon

After determining the EphB2/ephrin-B1 and MCP-1 expression status and macrophage infiltration, MAPK/ERK pathway activity was measured in the colons of KKAy and C57BL mice by IHC. ERK activation triggered inflammatory cytokine secretion by adipose tissue, which was followed by ephrin-B1 downregulation. This upregulated MCP-1 via ERK1/2 activation, in turn promoting macrophage recruitment (23). The colon of KKAy mice showed significantly more ERK1/2 expression in the upper half of crypts than that of C57BL mice (Fig. S1A). The ERK1/2 immunoreactive score (IRS) was significantly higher in KKAy than C57BL mice (7.11 vs. 2.83, P<0.001) (Fig. S1B).

### Expression of EphB2 and ephrin-B1 in human colon

**Figure 3.**
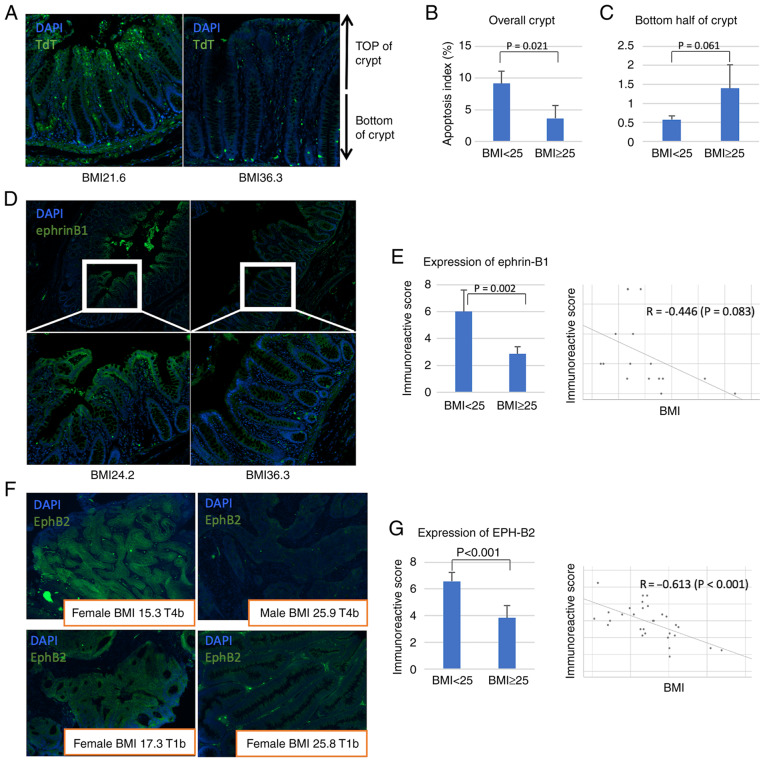
TdT-mediated dUTP nick-end labelling, and ephrin-B1/EphB2expression analysis in the human colon. (A) Representative images of fluorescent TUNEL staining of normal mucosa in a normal-weight (BMI 21.6) and an obese (BMI 36.3) individual. Magnification, ×100. (B) Apoptosis index in the overall crypt in obese (BMI ≥25) and healthy (BMI <25) individuals. (C) Apoptosis index in the bottom half of the crypt of obese (BMI ≥25) and healthy (BMI <25) individuals. (D) Representative immunohistochemistry images of ephrin-B1 stained normal mucosa from a non-obese (BMI 24.2) and an obese (BMI 36.3) individual. Magnification, ×40 (above) and ×100 (below). (E) Association between the immunoreactive scores of ephrin-B1 and the BMI. (F) Representative immunohistochemistry images of T4b and T1b tumours from non-obese and obese patients after EphB2 staining. Magnification, ×100. (G) Association between the immunoreactive scores of EphB2 and the BMI. Error bars indicate 95% confidence intervals. TdT, terminal deoxynucleotidyl transferase; dUTP, deoxyuridine triphosphate; C57BL, C57BL/6JJcl mice; KKAy, C57BL/6JJcl-derived KK-Ay/TAJcl mice.

Next, the results obtained from the obesity mouse model were validated in human samples. The patient characteristics are detailed in the Table II. TUNEL assay revealed that the number of apoptotic cells in normal mucosa was significantly lower in patients with BMI >25 compared with patients with BMI<25 (Fig. 3A). Furthermore, the apoptotic cells in the mucosa of obese patients were more frequently found in the base of the crypt, similar to the mouse model (Fig. 3B and C).

Ephrin-B1 was expressed at lower levels in the normal colon mucosa of patients with a high BMI (Fig. 3D); the IRS of ephrin-B1 was significantly lower in patients with a high BMI than that in patients with a low BMI (6.00 in BMI <25 vs. 2.86 in BMI ≥25, P=0.002, Mann-Whitney U test; Fig. 3E). On the other hand, EphB2 was expressed at higher levels in tumours of patients with a lower BMI (Fig. 3F). Moreover, the IRS of EphB2 was significantly higher in patients with a lower BMI (6.58 in BMI <25 vs. 3.83 in BMI ≥25, P<0.001, Mann-Whitney U test; Fig. 3G). These results suggest that obesity results in dysregulation of EphB2 and ephrin-B1 expression, promoting obesity-related cancer development and progression.

The EphB2 level was analysed according to the degree of tumour differentiation (poorly differentiated, p/d; well-differentiated, w/d; and moderately differentiated, m/d) to evaluate the association between tumour malignancy and EphB2. However, there was no significant difference in the degree of differentiation among w/d, m/d, and p/d tumours (IRS=6.0, 5.5, and 6.25 in the w/d, m/d, and p/d groups, respectively, P=0.896, Kruskal-Wallis test) (Fig. S2).

## Discussion

The association between obesity and colorectal carcinogenesis has been demonstrated by numerous epidemiological studies. Our findings demonstrate that obesity and obesity-induced inflammation promote colon cancer development and progression. Our findings also suggest that repulsive Eph-ephrin interactions play a critical role in obesity-associated colorectal cancer. In obesity, the ephrin-B1 expression in normal colon mucosa is downregulated, leading to decreased cell apoptosis and carcinogenesis. The obesity-induced secretion of inflammatory cytokines, including MCP-1, drive carcinogenesis by downregulating ephrin-B1 expression. Obesity also results in EphB2 downregulation, leading to the more rapid cancer development and progression observed in obese mice and humans. Our study also offers new insight into how obesity promotes colorectal tumourigenesis and enhances cancer progression.

It was found that ephrin-B1 expression at the top of the crypt was downregulated in obesity and that the dysregulated EphB2/ephrin-B1 signalling may disrupt cell apoptosis and carcinogenesis. Previous studies have suggested a correlation between Eph-ephrin signalling and colorectal cancer. In the colon of *Apc*^Min/+^ mice, Eph-B expressing tumour cells expanded laterally, forming additional crypt structures that replaced the normal epithelium, and resulted in the apparent compartmentalisation of Eph-B-positive tumour cells and ephrin-B-positive normal cells (9,24,25). Furthermore, Cortina *et al* demonstrated that in ephrin-B-deficient mice, *Apc*-mutant cells repopulated the normal mucosa due to the lack of repulsive interaction between Eph-B and ephrin-B (15). As a result, the progression of adenomas was suppressed by Eph-ephrin repulsive signals. In obesity, reduction in ephrin-B1 levels may lead to apoptosis inhibition in the crypt-villus axis and subsequent development and progression of adenoma. Our immunohistological findings support the premise that obesity promotes adenoma and carcinoma development by the disruption of Eph-ephrin signalling.

Eph-ephrin signalling also plays a critical role in the progression of colorectal cancer. The current study demonstrated that KKAy mice had larger tumours than C57BL/6 mice and that Eph downregulation is a crucial step in the progression of obesity-associated colorectal cancer. Clevers and Batlle suggested that Eph-B expression was disrupted in a subset of tumour cells and that, in the absence of Eph-B, tumour progression was accelerated, resulting in the development of highly aggressive colorectal adenocarcinomas (14). EphB2 downregulation has been associated with poor prognosis in various human cancers, including colorectal cancer (26,27). It has been reported that mutations in repetitive sequences in the exon 17 of EphB2 are frequent in colon adenomas and colorectal carcinomas. In addition, hypermethylation of EphB2 promoter is frequently found in colorectal cancer patients (28). Another study has shown that DNA methylation is an important mechanism regulating gene expression in mice receiving a high fat diet (29). Taken together, these studies suggest that obesity results in EphB2 downregulation in colorectal cancer by promoting the methylation of its promoter. The association between obesity and EphB2 mutations should be analysed in future studies.

The relationship between Eph-ephrin signalling and obesity-associated carcinogenesis was also analysed. Decreased expression of ephrin-B1 associated with increased macrophage infiltration in the colon mucosa. Mori *et al* suggested a positive feedback regulation between inflammation, MCP-1, and ephrin-B1 (18). Obesity induces the secretion of inflammatory cytokines in adipose tissue, such as MCP-1 and tumour necrosis factor-α (TNF-α) via activation of the ERK, nuclear factor-κB (NFκB), and c-Jun N-terminal kinase (JNK) pathways, which contribute to ephrin-B1 downregulation. The disruption in ephrin-B1 expression results in MCP-1 upregulation via TNF-α-mediated ERK1/2 activation, which in turn promotes the recruitment of macrophages; macrophages further promote ephrin-B1 downregulation. This network is believed to also occur in the colon mucosa of obese individuals. In addition, previous studies suggested that mitogen-activated protein kinase/ERK (MAPK/ERK) signalling occurs downstream of ephrin-EphB signalling and that Eph signals suppress the ERK activity (30,31). These reports supports our results. The dysregulation of ephrin signals and activation of MAPK/ERK pathway can lead to the exacerbation of inflammation and carcinogenesis in the intestinal mucosa (23,31). However, evidence supporting an association between obesity, ephrin-Eph signalling, MAPK/ERK pathway and colorectal cancer development and progression is still lacking. Further studies are therefore needed.

**Figure 4.**
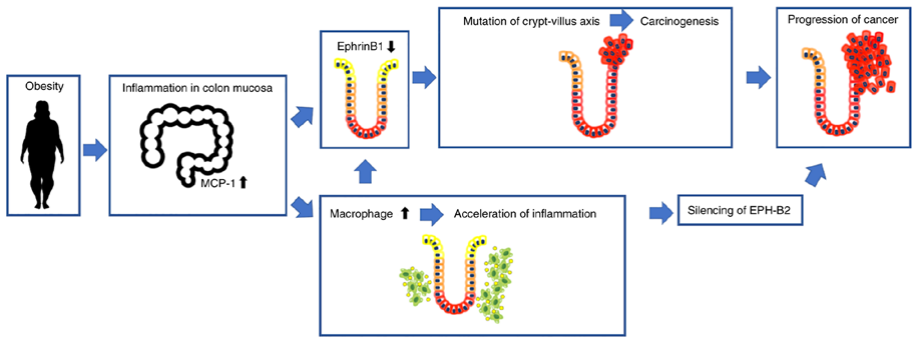
Schematic diagram illustrating the possible role of Eph-ephrin signalling and colon inflammation in obesity-associated carcinogenesis. Repulsive interactions occur between EphB2-positive cells at the bottom of the crypt and ephrin-B1-positive cells at the top of the crypt, and these interactions regulate the position of cells in the crypt. However, obesity-induced inflammation suppresses ephrin-B1 expression at the top of the crypt, disrupting the repulsive EphB2/ephrin-B1 interaction and promoting the migration of mutation-harbouring cells at the bottom of the crypt; these cells evade apoptosis and grow into adenoma. The expression of EphB2 is also downregulated in obesity, leading to obesity-associated colorectal cancer progression. MCP-1, monocyte chemoattractant protein-1; EPH-B2, ephrin-B2.

According to findings of this study, in normal colon mucosa, repulsive interactions occur between Eph-B2-positive cells at the bottom of the crypt and ephrin-B1-positive cells at the top of the crypt, and these interactions regulate the position of cells in the crypt (Fig. 4). However, obesity-induced inflammation results in ephrin-B1 downregulation at the top of the crypt, disrupting the repulsive EphB2/ephrin-B1 interaction and promoting the migration of mutation-harbouring cells at the bottom of the crypt; cells in the crypt evade apoptosis and eventually grow into adenoma. The expression of EphB2 is also downregulated by undetermined factors; hence, the repulsive Eph-ephrin interaction is fully lost, leading to obesity-associated colorectal cancer progression. A better understanding of the mechanism underlying obesity-associated cancer development and progression will allow for the discovery of therapeutic approaches. Anti-inflammatory therapy targeting macrophage infiltration or inhibiting the silencing of EphB2 may offer novel approaches for inhibiting obesity-associated colorectal cancer development and progression.

There were several limitations to this study. First, the obesity mouse model that was used may not fully reflect the acquired obesity. The KKAy mouse strain was developed by introducing the Ay mutation into the inbred KK strain of native Japanese mice (20), which leads to the development of congenital obesity. In addition, there are several confounding factors in the relationship between obesity and cancer development, such as diabetes, diet and the level of physical exercise. Although KKAy mice have been widely used in mouse model of obesity (32–34) further assessment using another obesity model mice, e.g. leptin-deficient ob/ob mouse or leptin receptor deficient db/db mouse, can validate our results. Second, obesity is only one of many lifestyle-related diseases; this study analysed obesity independently of other conditions. Since excess weight and obesity are closely linked with hypertension, hyperglycaemia, and hyperlipidaemia, which are collectively referred to as metabolic syndrome, it is important also to consider the effect of metabolic syndrome in relation to cancer as a whole. Third, MCP-1 expression and macrophage infiltration in human mucosa were not evaluated; therefore, further studies are needed to validate the present data. Fourth, downstream pathway markers activated by ephrinB1/EphB2 signal are not fully evaluated in this study. Further analysis such as an evaluation of phosphorylated transducer and activator of transcription-3 is needed to validate our findings. Finally, our investigations focused on EphB2 and ephrin-B1 expression in colorectal cancer; however, other ephrins, including Eph-B3 and Eph-B4, have also been implicated in colorectal carcinogenesis (10,12,16,35). A comprehensive analysis of the role of the ephrin family in obesity-associated colorectal cancer should be conducted.

In conclusion, the findings of this study suggest that obesity-induced inflammation results in the disruption in Eph-ephrin signalling and the crypt-villus axis, promoting oncogenesis. This study highlights the importance of Eph-ephrin signalling in obesity-associated colorectal cancer.

## Supplementary Material

Supporting Data

## Figures and Tables

**Table I.** Comparison of azoxymethane-induced colon carcinogenesis in KKAy mice and C57BL mice.

Parameter	C57/BL	KKAy	P-value
Number of mice	15	10	
Number of mice with tumours	2	10	<0.001
Number of tumours per a mouse	0.13±0.35	8.40±3.06	<0.001
Largest tumour diameter (mm)	0.47±1.36	7.40±5.70	<0.001

C57BL, C57BL/6JJcl mice; KKAy, C57BL/6JJcl-derived KK-A^y^/TAJcl mice.

**Table II.** Patient characteristics.

Characteristic	BMI <25	BMI >25
Tumour samples, n	32	16
Age, years ± SD	65.7±11.5	64.1±11.4
Sex, n		
Male	17	9
Female	15	7
Location, n		
Colon	26	11
Rectum	6	5
T stage, n		
T1,2	7	5
T3,4	25	11

BMI, body mass index.

## Data Availability

The datasets used and/or analysed during the current study are available from the corresponding author on reasonable request.
