# Supplementary material for: Role of EphB2/ephrin-B1 signalling in the development and progression of obesity-associated colorectal cancer
Source: Oncol Lett. 2022 Jul 19;24(3):316. doi: 10.3892/ol.2022.13436 (PMC9353875; doi:10.3892/ol.2022.13436)

Figure S1. ERK1/2 expression levels in the murine colon. (A) Representative immunohistochemical images of normal and ERK1/2-expressing mucosa of C57/BL and KKAy mice. Magnification, x200. (B) ERK1/2 immunoreactive scores in the upper half of the crypt of C57/BL and KKAy mice. C57/BL, C57BL/6JJcl mice; KKAy, C57BL/6JJcl-derived KK-A^{y}/TAJcl mice.

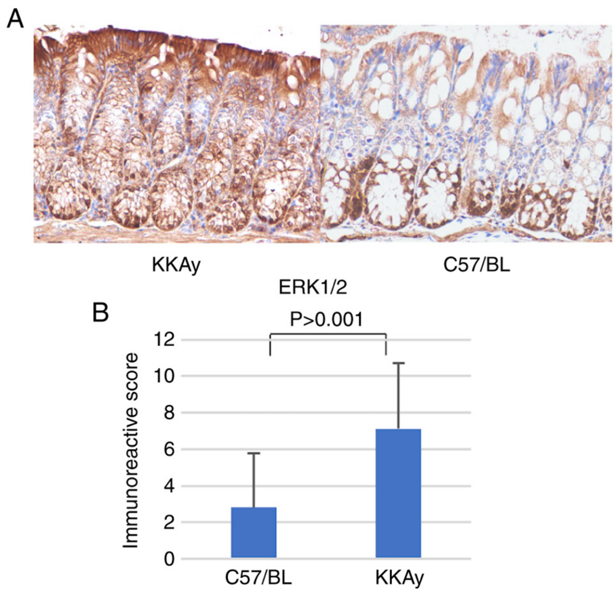

Figure S2. Association between EphB2 expression and tumour differentiation in the human colon. (A) Representative immunohistochemistry images of EphB2-stained in well-, moderately and poorly differentiated tumours. Magnification, x200. (B) Immunoreactive tumour EphB2 scores according to the degree of tumour differentiation. w/d, well-differentiated; m/d, moderately differentiated; p/d, poorly differentiated; Eph, ephrin.

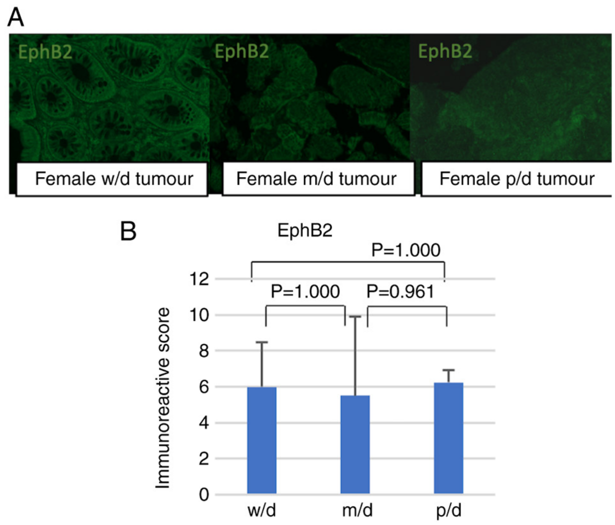

Supplement: Supporting Data [file Supplementary_Data.pdf]
